# Green Manure Addition to Soil Increases Grain Zinc Concentration in Bread Wheat

**DOI:** 10.1371/journal.pone.0101487

**Published:** 2014-07-07

**Authors:** Forough Aghili, Hannes A. Gamper, Jost Eikenberg, Amir H. Khoshgoftarmanesh, Majid Afyuni, Rainer Schulin, Jan Jansa, Emmanuel Frossard

**Affiliations:** 1 Institute of Agricultural Sciences, Department of Environmental Systems Science, Swiss Federal Institute of Technology (ETH) Zürich, Switzerland; 2 Paul Scherrer Institute (PSI), Radioanalytics Laboratory, Villigen, Switzerland; 3 College of Agriculture, Department of Soil Sciences, Isfahan University of Technology, Isfahan, Iran; 4 Institute of Terrestrial Ecosystems, Department of Environmental Systems Science, Swiss Federal Institute of Technology (ETH) Zürich, Switzerland; 5 Institute of Microbiology, Academy of Sciences of the Czech Republic, Prague, Czech Republic; Institute for Sustainable Plant Protection - C.N.R., Italy

## Abstract

Zinc (Zn) deficiency is a major problem for many people living on wheat-based diets. Here, we explored whether addition of green manure of red clover and sunflower to a calcareous soil or inoculating a non-indigenous arbuscular mycorrhizal fungal (AMF) strain may increase grain Zn concentration in bread wheat. For this purpose we performed a multifactorial pot experiment, in which the effects of two green manures (red clover, sunflower), ZnSO_4_ application, soil γ-irradiation (elimination of naturally occurring AMF), and AMF inoculation were tested. Both green manures were labeled with ^65^Zn radiotracer to record the Zn recoveries in the aboveground plant biomass. Application of ZnSO_4_ fertilizer increased grain Zn concentration from 20 to 39 mg Zn kg^−1^ and sole addition of green manure of sunflower to soil raised grain Zn concentration to 31 mg Zn kg^−1^. Adding the two together to soil increased grain Zn concentration even further to 54 mg Zn kg^−1^. Mixing green manure of sunflower to soil mobilized additional 48 µg Zn (kg soil)^−1^ for transfer to the aboveground plant biomass, compared to the total of 132 µg Zn (kg soil)^−1^ taken up from plain soil when neither green manure nor ZnSO_4_ were applied. Green manure amendments to soil also raised the DTPA-extractable Zn in soil. Inoculating a non-indigenous AMF did not increase plant Zn uptake. The study thus showed that organic matter amendments to soil can contribute to a better utilization of naturally stocked soil micronutrients, and thereby reduce any need for major external inputs.

## Introduction

Wheat is the third most-grown cereal after maize and rice, draws on the largest cropping area, and ranks first in global cereal trade [1]. It is the crop contributing most calories to large segments of the global human population. Yet, wheat-based diets do not provide sufficient zinc (Zn), which is a leading cause of widespread Zn deficiency [2]. Low grain Zn concentration is cause of low plant-availability of Zn in many soils on which wheat is produced [3], [4]. Efforts have been made to breed wheat cultivars efficient in acquiring Zn from soil and effective in (re-) translocating Zn to grains [5], [6]. Zinc supply from soil has, however, to be increased for such elite crop cultivars to become fully effective, which can be achieved via appropriate soil fertility management, such as application of mineral Zn fertilizer [7], addition of green manure [8], and/or a strengthening of nutrient acquisition traits, including symbioses of roots with naturally occurring and newly introduced arbuscular mycorrhizal fungi (AMF) [9]–[12].

Plants and probably also AMF take up Zn from the soil solution in form of free Zn^2+^ and ZnOH^+^ ions, or as Zn-organic ligand complexes [13], [14]. The depleted soil solution is replenished with Zn via desorption or dissolution from the soil matrix, followed by dispersion by diffusion [15]. Desorption from the soil matrix is controlled by soil pH, salinity, and clay, carbonate, and organic matter contents [15]. To promote Zn dissolution plants and microorganisms exude soil-acidifying protons and Zn-chelating low-molecular-weight organic compounds [16], [17]. These Zn mobilizing activities of roots and microorganisms are promoted under iron (Fe), Zn, and other nutrient deficiencies [18]. The most well known classes of exuded organic Zn ligands are carboxylates, amides, and non-proteinaceous amino acids ( =  siderophores) [19], [20]. A recent study on Zn uptake by wheat in the field [8] suggests that residues from the previous crop in crop rotations and thus possibly also addition of green manure to soil can raise the plant-available Zn in soil and promote Zn uptake in crop plants, mainly by contributing Zn-chelating dissolved organic carbon and amino acids.

Arbuscular mycorrhizal fungi, phylum Glomeromycota), because of their ubiquity and crucial involvement in plant nutrient acquisition and health, are a plant production-relevant group of root symbionts [21], [22]. Their hyphae form effective, large-distance conduits for the transport of temporarily and spatially heterogeneously available nutrients of low mobility in soil, such as phosphate (P) and Zn [23]. Crop residue or green manure addition to soil and sporadic rain and irrigation may create nutrient flushes that crop plants with root systems extended by extraradical AMF hyphae may much more effectively utilize [24]. Arbuscular mycorrhizal fungi, whether indigenous or deliberately introduced as inoculants, were repeatedly reported to benefit Zn uptake in crop plants [9], [10]. While the phenomenon of increased plant Zn uptake of mycorrhized as opposed to non-mycorrhized plants has already often been demonstrated [10], we still lack information about controlling factors [8] and the possible reinforcing effect of combined enrichment of soils with organic matter and mineral Zn fertilizer.

Here we report findings of a cross-factorial pot experiment on Zn nutrition of bread wheat involving the enrichment of calcareous soil with green manure of red clover and sunflower and inoculating plants with a non-indigenous AMF strain. For comparison and to study its interactions with the other experimental factors also a water-soluble mineral Zn fertilizer (ZnSO_4_) was applied. To compare the effect of all naturally occurring AMF and possibly associated other soil microorganisms, the soil of half of the experimental units was γ-irradiated and re-inoculated with an AMF-free microbial filtrate of a water suspension of non-sterilized soil. An AMF inoculation treatment was further included in an attempt to assess the potential of this rapidly growing technology and for controlling against non-AMF mediated biotic effects by the soil γ-irradiation and microbial re-inoculation procedure. We predicted that 1) green manure addition to soil improves Zn uptake by wheat, 2) green manure addition improves Zn uptake from mineral Zn fertilizer and 3) inoculation of wheat plants with a foreign AMF strain improves Zn uptake, particularly from soil whose indigenous AMF had been killed by γ-irradiation.

## Materials and methods

### Experimental design

A complete cross-factorial experiment with the factors green manure (3 levels: none, red clover, sunflower), mineral Zn fertilization (2 levels: none, ZnSO_4_), γ-irradiation (2 levels: non-irradiated; γ-irradiated and re-inoculated with soil microbes, except AMF), and AMF inoculation (2 levels: none, inoculated) was set up with each treatment replicated five times. The 120 experimental pots were arranged randomly on a glasshouse bench.

### Soil origin and physicochemical soil analyses

The soil was collected in September 2009 to a depth of 30 cm from an arable field at the Rudasht research station (32, 29/N, 52, 10/E, 1560 m above sea level), located 65 km south-east of Isfahan in central Iran [permission for soil sampling was given by the Isfahan Agricultural and Natural Resource Research Center and for its import to Switzerland by the Swiss Federal Department of Agriculture]. The soil was air-dried and passed through a 5 mm sieve, before being shipped to Switzerland, where 5 kg were further passed through a 2 mm sieve for physicochemical analyses.

This experimental soil was a Typic Haplocambid, according to the USDA soil taxonomy [25]. The field where the soil was sampled had been under agricultural use with periodical irrigation from a nearby river. The crop rotation prior to soil sampling comprised barley, wheat, sugar beet, and wheat, interrupted by dry fallow periods. The climate in central Iran is semi-arid with a long-term mean annual temperature of 16.8°C, a mean annual rainfall lower than 100 mm, and a potential annual evaporation higher than 2000 mm [26].

The soil texture was a silty clay loam based on the measurements by the hydrometer method [27] and according to the USDA soil classification scheme [6]. The pH was 7.9 and the electrical conductivity (EC) 23 dSm^−1^, as determined by the procedure of Rhoades [28], using an ISE pH meter 720A (Mettler Toledo LLC, Columbus, OH, USA) and an E- 518 conductometer (Metrohm Herisau, Switzerland), respectively. The soil was low in organic carbon [5 g (kg soil) ^−1^] and nitrogen [0.6 g (kg soil) ^−1^], as estimated by the methods of Walkley and Black [29] and on a Carlo Erba flash combustion CN-analyzer (NA1500, Carlo Erba, Milano, Italy), respectively. The soil contained 300 mg CaCO_3_ (kg soil)^−1^ as determined after neutralization with hydrochloric acid and back titration with sodium hydroxide [30].

The total Zn concentration of the soil was 80.2 mg Zn (kg soil)^−1^, according to energy dispersive X-ray fluorescence spectrometry (XRF) measurements on a Spectra X-Lab 2000 instrument (SPECTRO Analytical Instruments GmbH, Kleve, Germany). Potentially plant-available Zn was extracted from 25 g of soil, using 50 ml of 0.05 M diethylene triamine-penta-acetic acid (DTPA), adjusted to pH 7.2 [31], and measured on an Agilent 7500 C inductively coupled plasma mass spectrometer (ICP-MS, Agilent Technologies, Santa Clara, California, USA). The amount of DTPA-extractable Zn was 0.45 mg Zn (kg soil)^−1^.

### Partial soil desalination, γ-irradiation and fertilization

Preliminary tests showed that wheat growth on the untreated soil was severely impaired by salinity stress (Forough Aghili et al., unpubl. data). Therefore, we flushed the soil in five rounds, with a volume ratio of 1∶1 soil:deionized water each prior to the experiment. This treatment can be considered comparable to repeated flood irrigation, used by farmers at the field site prior to sowing. Soil washing lowered the EC from 23 to 6.7 dS m^−1^, which is close to the threshold value of 5.9 dS m^−1^ for salinity-induced growth depression in wheat [32]. Flushing with water also slightly decreased the soil pH from 7.9 to 7.7. Analyses of the leachate indicated that large amounts of sodium, chlorine and sulfur had been removed, but virtually none of the total soil Zn (data not shown). The DTPA-extractable concentration of Zn in the soil even increased slightly from 0.45 to 0.47 mg Zn (kg soil)^−1^. No spores of AMF could be retrieved from the leachate. The partially desalinated soil was again air-dried and sieved to a particle size of ≤5 mm.

Half of the soil was γ-irradiated with a dose of 25–75 kGy using a ^60^Co source (Studer-Hard, Däniken, Switzerland, http://www.leoni-irradiation-services.com) in order to kill the indigenous soil microbes. To reintroduce soil microbes, but not AMF, 50 ml of a filtrate of a 2.5% (w:v) soil water suspension was added per kilogram soil. This microbial filtrate was prepared by passing the soil suspension twice through Whatman No 1 filter papers, which size-excluded AMF propagules. This procedure was recommended by Thompson [33] for studying the effect of AMF on Zn nutrition and is also utilized in many comparable studies to analyze the relative effect of different soil microorganisms on plant nutrition [34], [35]. Indeed, soil γ-irradiation is currently the only way to create a control treatment, free of naturally occurring AMF, since mycorrhiza-defective mutants of wheat are not available [36].

The flushed soils (non-irradiated and γ-irradiated) received 400 mg K, 200 mg P, and 80 mg Fe per kilogram soil in the form of finely ground K_2_SO_4_, CaHPO_4_ and FeSO_4_ 7H_2_O salts.

### Preparation and application of ^65^Zn-labeled green manure

We chose red clover (*Trifolium pratense L.*) and sunflower (*Helianthus annuus* L.) for the production of experimental green manures, because they are also often grown before wheat in the region of Isfahan [8]. Furthermore, Soltani et al. [8] found that compared to other possible green manure plants, red clover and sunflower introduced significant amounts of dissolved organic carbon into the soil solution, which was probably important for Zn solubilization.

Seeds of clover and sunflower were surface sterilized in 15% H_2_O_2_ for 15 min, rinsed with distilled water and germinated on moistened filter papers for two days. The most vigorously growing seedlings were transferred to a combined sand bed-hydroponics system, which consisted of compartmented seedling trays filled with silica sand of a particle size of 0.7–1.2 mm, partially immersed in a full-strength Hoagland nutrient solution [37]. After 28 days, the nutrient solution was exchanged for one that contained radioactive ^65^Zn for an additional 26 days of vegetative plant development. While 30 liters of Hoagland nutrient solution were labeled with 5.55 MBq of ^65^Zn for about 400 red clover plantlets, 40 l of Hoagland nutrient solution were labeled with 9.25 MBq of ^65^Zn for about 140 sunflower plantlets. The ^65^Zn radiotracer was added as carrier-free ZnCl_2_, dissolved in weak HCl (Amersham plc, GE Healthcare, Little Chalfont, UK). The photoperiod in the glasshouse was set to 14 h, the minimal light intensity to 12 kLux, the temperature to 22°C during the day and 16°C at night, and the relative air humidity was allowed to vary between 30 and 40%.

At harvest, the entire shoots of red clover and only the leaf blades of sunflower, without petioles and stems, were collected, air-dried and finely ground to powder. The total tissue C and N concentrations were determined in 4 mg tissue samples using the above-mentioned CN-analyzer. The total P and Zn concentrations were measured in 100 mg subsamples of each of the green manures, using the above-mentioned ICP-MS after incineration at 550°C for 6 h and extracted with 3 ml of hot 14.4 M nitric acid (HNO_3_). The concentration of radioactive ^65^Zn in the green manures was measured by γ-spectrometry in 300 mg subsamples using IGC2 high purity germanium detectors (ORTEC Advanced Measurements Technologies Inc, Oak Ridge, Tennessee, USA). Selected characteristics of both green manures are presented in Table 1.

**Table 1:** Mineral nutrient concentrations of the two types of applied green manure prepared from red clover and sunflower.

	Red clover	Sunflower
	(Mean ± SE)	(Mean ± SE)
**N mg (g DM** * **)^−1^**	43.3±0.6	58.4±0.4
**C/N (mass ratio)**	9.1±0.1	6.3±0.1
**P mg (g DM)^−1^**	5.0±0.1	7.3±0.1
**Zn μg (g DM)^−1^**	45.6±0.6	104.4±2.4
**^65^Zn kBq** ¶ **(g DM)^−1^**	6.9±0.02	15.8±0.1

* DM: dry matter.

¶ Radioactivity at the time of harvest of the experiment.

The means and associated standard errors (SE) of three replicate subsamples of the nitrogen (N) concentration, carbon (C) to N mass ratio, phosphorus (P) and zinc (Zn) concentrations, and ^65^Zn concentration are listed.

The green manures (Table 1) were homogenously mixed into the soils at a dosage of 4 g dry matter per kilogram of dry soil. This dosage corresponds approximately to an addition of 18 tons of dry matter per hectare considering a soil bulk density of 1.5 t m^−3^ and incorporation to a soil depth of 30 cm.

### Mineral Zn fertilization

Half of the soil in each green manure × irradiation treatment combination was fertilized with water soluble Zn in the form of ZnSO_4_ at a dosage of 4.7 mg Zn (kg soil)^−1^, corresponding approximately to 19 kg Zn ha^−1^.

### AMF inoculation

Finally, half of all pots in each combination of the above treatments were inoculated with approximately 250 spores of a pot culture of the Swiss isolate BEG155 of the AMF species *Claroideoglomus claroideum* [38]. The spores of the inoculum were extracted by wet-sieving and decanting [39] and directly pipetted onto the roots of the transplanted wheat seedlings. This careful spore isolation and inoculation procedure prevented co-inoculation of many other microbes and introduction of organic matter in the form of root pieces, which would have been the case with so-called whole inoculum { =  growth substrate, containing fungal spores, hyphae and colonized root fragments [40]}.

Data on AMF root colonization were not systematically collected in this experiment, since microscopic and molecular genetic quantification in a previous experiment with the same wheat cultivar and soil clearly showed a high infection pressure by the naturally occurring AMF (Forough Aghili et al., unpublished data). However, successful colonization of the roots by the AMF isolate BEG155 and by naturally occurring AMF were confirmed in a subset of all experimental treatments by quantitative polymerase chain reaction as in the previous study and additionally by phylogenetic sequence analysis of a 1.6 kb-long ribosomal DNA amplicon of the AMF colonizing the roots of a subset of all experimental units (Anouk Guyer et al., unpublished data).

### Planting, growth conditions, harvest and mineral nutrient analyses

Wheat kernels {*Triticum aestivum* cv. Kavir, a cultivar widely grown in Iran [41]} were surface-sterilized with 15% H_2_O_2_ for 15 min and pre-germinated on moistened filter paper for three days prior to transplantation. Four seedlings were planted into each pot with 550 g soil and later thinned to the most vigorously growing two individuals. Nitrogen in the form of NH_4_NO_3_ was applied weekly, six times, amounting to 600 mg N (kg soil) ^−1^ until tillering.

The climatic conditions in the glasshouse were set to a photoperiod of 14 h with a minimum light intensity of 10 kLux, a 22/17°C day/night air temperature regime, and 40–45% relative air humidity. Soil moisture was kept at about 70% soil water holding capacity by daily watering to weight.

Harvest took place at grain maturity, 125 days after planting. The total weight of the grains and straw of the two plants in each pot was determined after drying to constant weight at 60°C for 72 h. Subsamples of 200 mg of finely ground wheat straw and grain were incinerated in porcelain crucibles at 550°C for 6 h and the ashes dissolved in 2 ml of boiling 13 M HNO_3_. Zinc concentrations were measured using ICP-MS. Measurement accuracy and precision were ensured by regular inclusion of a certified plant reference standard in the analyses (hay powder IAEA-V-10, 24 mg Zn kg^−1^) and by running blank samples with each sample batch. The concentration of radioactive ^65^Zn was measured in the grains and straw by γ-spectrometry as described for the green manure above. Zinc uptake into roots could not be determined because it was not possible to recover the roots quantitatively from the clay-rich soil. Soil pH and the concentrations of total N and DTPA-extractable soil Zn were measured at the end of the experiment, using the above-described analytical procedures.

### Calculations and data analyses

#### Plant Zn uptake from green manure, soil and mineral Zn fertilizer

Plant Zn uptake was calculated as the product of Zn concentration and biomass for both the grain and straw fractions and summed to obtain the amount of Zn contained in the entire aboveground biomass. All radioactivity measurements were related to the harvest time, taking the radioactive decay into account (^65^Zn half-life time: 243.9 d). The percentage of Zn in the aboveground biomass derived from the green manure [Zn_dgm_ (%)] was calculated as:
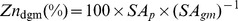
(1)where SA_p_ [Bq (mg Zn^−1^)] is the specific activity (SA) of Zn in the aboveground biomass and SA_gm_ the specific activity of Zn in the added green manure. The specific activity of Zn in the plant was calculated as:
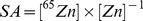
(2)where [^65^Zn] stands for the concentration of radioactivity per kilogram of plant material [Bq (kg DM)^−1^] and [Zn] stands for the concentration of total Zn in the plant [mg Zn (kg DM)^−1^].

Using the equations 1 and 2, the amount of Zn taken up to the aboveground biomass from green manure [Zn_dgm_, μg Zn (kg soil)^−1^] was calculated as:
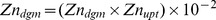
(3)where Zn_upt_ is the amount of Zn taken up to the aboveground plant biomass per kilogram of soil [μg Zn (kg soil)^−1^].

Knowing Zn_dgm_, it is possible to calculate the percentage of Zn recovered by the aboveground plant biomass from the added green manure [Zn_rec_gm_ (%)]:
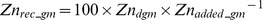
(4)where Zn_added_gm_ is the amount of Zn added with the green manure [μg Zn (kg soil)^−1^].

Knowing Zn_dgm_, it is also possible to calculate the amount of Zn in the plant that is derived from non-labeled Zn sources in soil, i.e. in the non-fertilized soil from the native soil Zn pool and in the mineral Zn-fertilized soil from the native soil Zn pool as well as the applied ZnSO_4_ [Zn_dsoil_, μg Zn (kg soil)^−1^]:
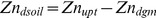
(5)


#### Statistical analyses

The results were analyzed by fixed factor four-way or three-way analyses of variance (ANOVA) with two levels (γ-irradiated and non-irradiated) of ‘soil γ-irradiation’, three levels (none, red clover and sunflower) of ‘green manure addition’, two levels [0 and. 4.7 mg Zn (kg soil)^−1^] of ‘mineral Zn fertilization’, and two levels (with and without inoculation with *C. claroideum*) of ‘AMF inoculation’. The interaction terms were sequentially removed from the initially complete ANOVA model when not significant (backward elimination). In addition, a one-way ANOVA was carried out on the saved residuals of a three-way ANOVA with the factors ‘soil γ-irradiation’, ‘mineral Zn fertilization’ and ‘AMF inoculation’ in order to specifically analyze the effect of green manure addition. Compliance of data dispersion with ANOVA model assumptions was verified using Shapiro-Wilcoxon's normality and Levene's equal variance tests. If necessary, data were log_10_ or squareroot transformed. All statistical analyses were carried out using the software package SPSS version 17 (SPSS Inc., Chicago, Ill inois, USA). Means of factor levels were compared by least-significant-difference (LSD) tests at the significance level of p<0.05. The figures were prepared in SigmaPlot version 12 [Systat Software Inc. (SSI), San Jose, California, USA].

## Results

### Plant biomass and grain nitrogen concentration

**Figure 1:**
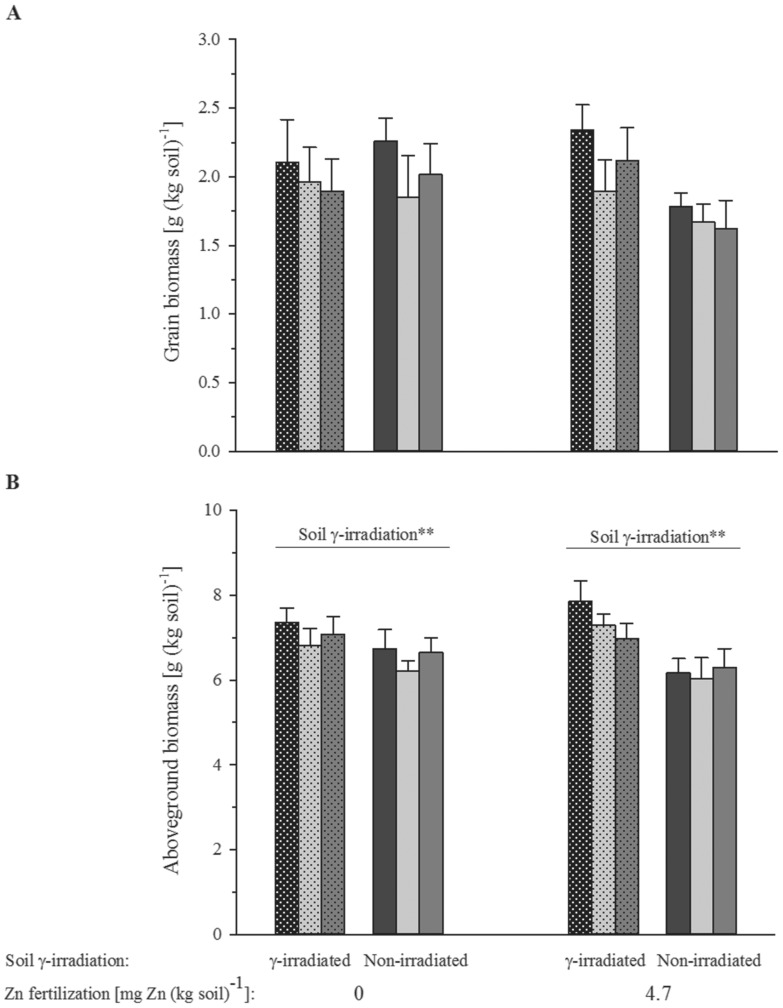
Grain yield (A) and aboveground biomass (B) of bread wheat grown in a calcareous soil. Black bar filling denotes the treatments without any added green manure, light grey bar filling the treatments in which the soil was amended with green manure of red clover, and dark grey bar filling those treatments in which green manure of sunflower was added to the soil. Bar hatching highlights the treatment missing the naturally occurring arbuscular mycorrhizal fungi after γ-irradiating the soil. Bars show mean values and associated standard errors of five experimental replicates. The significances (**, p<0.01) of soil γ-irradiation from a four-factorial analysis of variance are indicated. For full statistical details see Table 2.

The grain yield of the experimental wheat plants was not significantly affected by any of the treatments (Figure 1A, Table 2), but the large variance may have concealed a similar γ-irradiation effect as on the total aboveground biomass (Figure 1B). The total aboveground biomass ( =  straw + grains) was significantly larger for the wheat plants grown in γ-irradiated than non-irradiated soil (F_1,96_ = 10.45, p = 0.002). Inoculation with *C. claroideum* had no significant effect on aboveground biomass production (Table 2).

**Table 2:** Results of fixed factor four-way analyses of variance (ANOVA) on plant and soil parameters.

ANOVA model	Grain biomass		Aboveground biomass		Grain N concentration		Grain Zn concentration		Aboveground Zn concentration		Zn uptake into grains	
Source of variance	F value	df	F value	df	F value	df	F value	df	F value	df	F value	df
**Full model**	0.29^ns^	23	2.47*	23	4.45***	23	5.92***	23	8.91***	23	2.78**	23
**Green manure (A)**	0.33^ ns^	2	0.26^ns^	2	31.41***	2	13.48***	2	13.71***	2	1.98^ns^	2
**Zn fertilization (B)**	0.19^ ns^	1	0.12^ns^	1	0.09	1	50.96***	1	125.23***	1	40.52***	1
**Soil γ-irradiation (C)**	0.20^ ns^	1	10.45**	1	10.04	1	2.99^ns^	1	9.15**	1	0.17^ns^	1
**Inoculation (D)**	0.76^ ns^	1	0.13^ns^	1	2.97	1	1.95^ns^	1	1.04^ns^	1	0.95^ns^	1
**A×B**	r	2	r	2	R	2	12.71**	2	6.24**	2	12.71***	2
**A×C**	r	2	0.61^ns^	2	0.98^ns^	2	3.36*	2	4.41*	2	1.22^ns^	2

ANOVA model	Zn uptake into aboveground biomass		Zn_dgm_		Zn_rec_gm_		Zn_dsoil_		DTPA-extractable soil Zn	
Source of variance	F value	df	F value	df	F value	df	F value	df	F value	df
**Full model**	4.26***	23	5.95***	15	8.61***	15	6.15***	23	16.40***	23
**Green manure (A)**	4.13*	2	39.92***	2	72.25***	2	3.50*	2	85.22***	2
**Zn fertilization (B)**	53.49***	1	21.23***	1	22.31***	1	77.13***	1	168.84***	1
**Soil γ-irradiation (C)**	0.01^ns^	1	5.21*	1	8.85**	1	1.14^ns^	1	0.64^ns^	1
**Inoculation (D)**	2.34^ns^	1	8.25**	1	4.82*	1	1.26^ns^	1	2.10^ns^	1
**A×B**	0.49^ns^	2	2.12^ns^	2	0.13^ns^	2	2.70^ns^	2	2.17^ns^	2
**A×C**	0.44^ns^	2	0.63^ns^	2	4.64*	2	2.95^ns^	2	0.54^ns^	2

DTPA: diethylene triamine-penta-acetic acid.

na: not applied, r: removed from the statistical model, ns: not significant, *, p<0.05; **, p<0.01; ***, p<0.001.

The measurements of the plant parameters {grain biomass, total above ground biomass, grain N concentration, grain Zn concentration, aboveground Zn concentration, Zn uptake to grains, Zn uptake to the aboveground biomass, Zn derived from green manure in the aboveground biomass [Zn_dgm_], proportion of the Zn recovered in the above ground biomass [Zn_rec_gm_] of the Zn added with the green manure Zn derived from the soil and fertilizer in the aboveground biomass [Zn_dsoil_]}and soil parameter (DTPA-extractable Zn from soil) were made at grain maturity. Effect sizes of the experimental factors and their significant interactions are indicated as F-values alongside with the statistical significance level. The interactions of the factors A×D, B×C, B×D, C×D, A×B×C, A×B×D, A×D×C, B×C×D, A×B×C×D were not significant in any of the 11 ANOVAs and thus these results are not listed.

**Figure 2:**
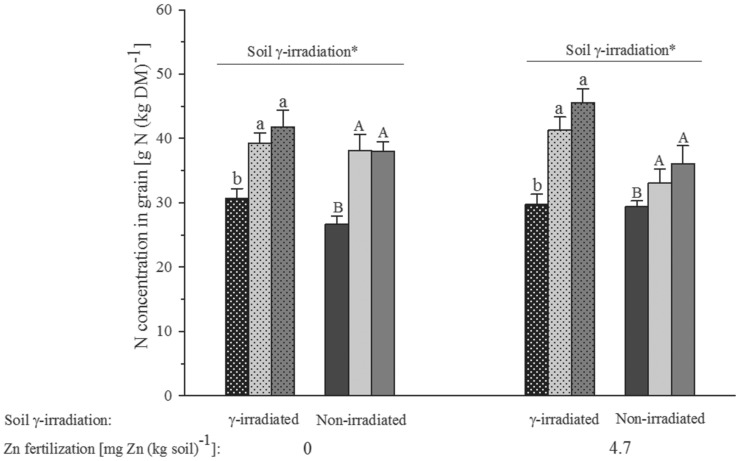
Grain nitrogen (N) concentration of wheat grown in a calcareous soil. Bar filling denoting the different green manure addition treatments is the same as in Figure 1. Bars show mean values and associated standard errors of five experimental replicates. The significances (*, p<0.05) of the effect of soil γ-irradiation from a four-factorial analysis of variance are shown. Different letters indicate statistical differences of separate least significant difference tests at p<0.05 for the different green manure addition treatments within the combinations of soil γ-irradiation and mineral Zn fertilization. For full statistical details see Table 2.

Both types of green manure significantly increased grain N concentration (F_2,70_ = 31.41, p<0.001, Table 2), which ranged between 30 and 45 g N (kg DM)^−1^ in the plants grown on γ-irradiated soil and between 25 and 37 g N (kg DM)^−1^ in the plants grown on non-irradiated soil (Figure 2). Soil γ-irradiation also led to a significant increase in grain N concentrations (F_1,70_ = 10.04, p = 0.003, Table 2). Inoculation with *C. claroideum* and ZnSO_4_ addition had no significant effects on grain N concentration (Table 2).

### Grain Zn concentration and Zn uptake to grains and aboveground biomass

**Figure 3:**
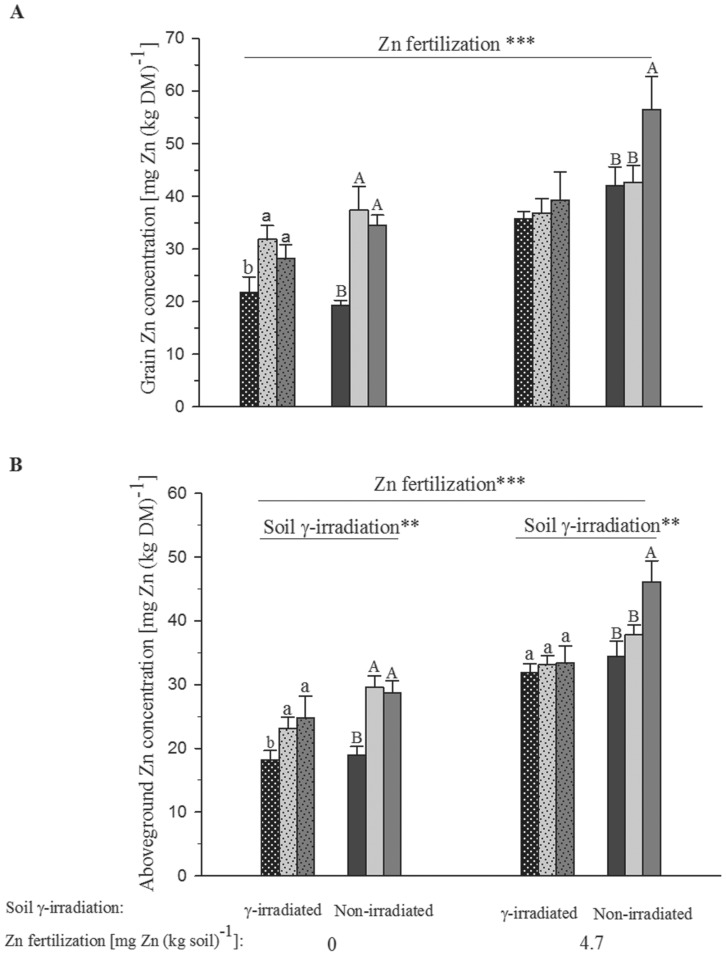
Grain (A) and aboveground (B) zinc (Zn) concentrations of wheat grown in a calcareous soil. Bar filling denoting the different green manure addition treatments is the same as in Figure 1. Bars show mean values and associated standard errors of five experimental replicates. The significances (**, p<0.01; ***, p<0.001) of the effect of mineral Zn fertilization, soil γ-irradiation of fixed factor four-way analyses of variance are shown. Different letters indicate statistical differences of separate least significant difference tests at p<0.05 for the different green manure addition treatments within the combinations of soil γ-irradiation and mineral Zn fertilization. For full statistical details see Table 2.

Grain Zn concentration increased significantly with application of mineral Zn fertilizer (F_1,70_ = 50.96, p<0.001) and green manure (F_2,70_ = 13.48, p<0.001), but was not affected by inoculation of *C. claroideum* and soil γ-irradiation (Figure 3A, Table 2). The lowest grain Zn concentration [20±1.64 mg Zn (kg DM)^−1^] was measured in the treatment with neither mineral Zn fertilization, nor addition of green manure, while the highest grain Zn concentration [54±2.32 mg Zn (kg DM)^−1^] was found in the treatment with combined application of ZnSO_4_ fertilizer and green manure of sunflower (Figure 3A).

The highest Zn concentration in the aboveground biomass [46.1±1.43 mg Zn (kg DM)^−1^] was found in plants grown on non-irradiated soil amended with green manure of sunflower in combination with ZnSO_4_ (Figure 3B). The lowest aboveground Zn concentration was observed in plants grown on γ-irradiated soil that had neither been fertilized with mineral Zn, nor amended with green manure (Figure 3B). Application of mineral Zn fertilizer reduced the differences between the aboveground biomass Zn concentrations of those plants that had received green manure and those that had not, resulting in a statistically significant interaction between green manure addition and mineral Zn fertilization (F_2,96_ = 6.24, p<0.01; Table 2). Inoculation with *C. claroideum* had no significant effect on the concentration of Zn in the aboveground biomass (Table 2).

The amount of Zn taken up from the native soil Zn pool and mineral Zn fertilizer that was allocated to grains ranged between 46 and 88 µg Zn (kg soil)^−1^, while the total amount of Zn accumulated in the aboveground biomass ranged between 117 and 367 µg Zn (kg soil)^−1^ (Table 3). The lowest values were again observed in the absence of mineral Zn fertilization and green manure addition, while the highest values were observed after addition of mineral Zn fertilizer and green manure of sunflower. Adding green manure to the soil tended to increase Zn accumulation in the grains, when no mineral Zn fertilizer had been applied, but this effect was not statistically significant (Table 2). Inoculation with *C. claroideum* had no significant effect on Zn uptake to the grains (Table 2).

**Table 3:** Effects of green manure addition and ZnSO_4_ application to soil, soil γ-irradiation and inoculation of a non-indigenous arbuscular mycorrhizal fungus (AMF) on plant and soil parameters.

Green manure addition	Soil γ-irradiation	Zn added with ZnSO_4_	Zn added with green manure	AMF inoculation	Zn uptake into aboveground biomass	Zn_dgm_	Zn_dsoil_	DTPA-extractable soil Zn
		μg Zn (kg soil)^−1^			μg Zn (kg soil)^−1^	μg Zn (kg soil)^−1^		μg Zn (kg soil)^−1^
**None**	**γ-irradiated**	0	0	no	159.3±12.4		159.3±12.4	450± 20
	**γ-irradiated**	0	0	yes	127.6± 6.9		127.6± 6.9	440± 32
	**Non-irradiated**	0	0	no	117.4± 13.2		117.4± 13.2	460± 35
	**Non-irradiated**	0	0	yes	127.2± 10.4		127.2± 10.4	480± 42
	**γ-irradiated**	4700	0	no	251.6± 16.6		251.6± 16.6	2150± 100
	**γ-irradiated**	4700	0	yes	227.3± 14.3		227.3± 14.3	1960± 93
	**Non-irradiated**	4700	0	no	206.6± 13.2		206.6± 13.2	2180± 110
	**Non-irradiated**	4700	0	yes	228.4± 27.5		228.4± 27.5	1990± 160
**Red clover**	**γ-irradiated**	0	183	no	152.9± 21.4	5.56± 0.59	147.4± 21.3	1220± 100
	**γ-irradiated**	0	183	yes	156.9± 25.3	5.86± 1.03	151.1± 19.6	1500± 120
	**Non-irradiated**	0	183	yes	205.0± 18.6	8.52± 0.35	196.5± 18.4	2010± 70
	**Non-irradiated**	0	183	yes	176.0± 23.8	6.10± 0.42	169.9± 20.2	2430± 110
	**γ-irradiated**	4700	183	no	224.0± 34.1	4.51± 0.39	219.5± 34.1	5050± 120
	**γ-irradiated**	4700	183	yes	243.9± 10.6	4.93± 0.38	239.0± 12.6	3550± 74
	**Non-irradiated**	4700	183	no	202.9± 25.2	5.32± 0.52	197.6± 23.8	4430± 32
	**Non-irradiated**	4700	183	yes	226.7± 22.4	6.11± 0.54	220.6± 21.5	3950± 92
**Sunflower**	**γ-irradiated**	0	418	no	227.9± 39.6	10.32± 1.39	217.6± 31.4	3310± 66
	**γ-irradiated**	0	418	yes	128.9± 11.4	8.38± 0.96	120.6± 22.6	1750± 120
	**Non-irradiated**	0	418	no	189.9± 14.3	11.05± 0.87	178.9± 16.4	3230± 86
	**Non-irradiated**	0	418	yes	174.9± 30.2	9.45± 1.61	165.5± 20.7	1950± 33
	**γ-irradiated**	4700	418	no	258.9± 25.8	8.44± 0.64	250.5± 41.3	3850± 82
	**γ-irradiated**	4700	418	yes	205.8± 20.4	5.94± 0.75	200.0± 19.5	4990± 36
	**Non-irradiated**	4700	418	no	367.0± 40.2	8.5± 0.64	358.5± 37.4	4540± 120
	**Non-irradiated**	4700	418	yes	227.9± 24.5	5.97± 0.80	222.0± 26.2	4680± 130

DTPA: diethylene-triamine-penta-acetic acid

Mean values and associated standard errors of five experimental replicates of Zn uptake to the aboveground biomass, Zn in the aboveground biomass derived from green manure (Zn_dgm_), Zn derived from soil and fertilizer (Zn_dsoil_) and DTPA-extractable Zn from soil at grain maturity are listed.

### Zn derived from green manure and both, soil and mineral Zn fertilizer, in the aboveground biomass

**Figure 4:**
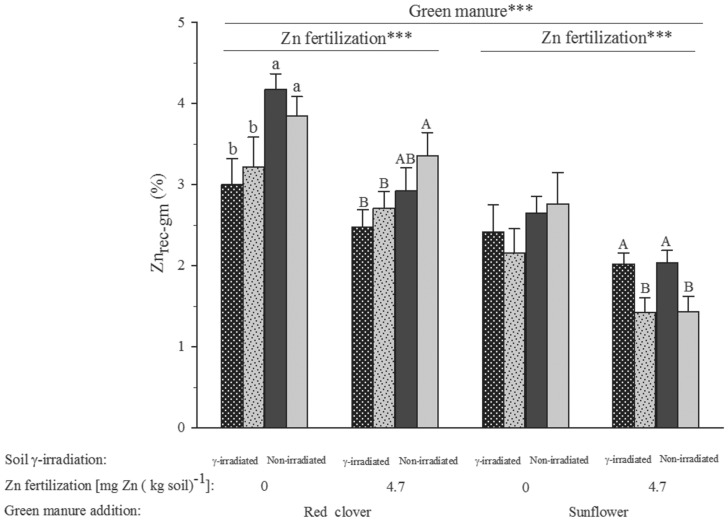
Percentage of zinc (Zn) recovered from green manure (Zn_rec-gm_) in the aboveground wheat biomass. Black bar filling denotes treatments with arbuscular mycorrhizal fungus (AMF) inoculation and light grey filling treatments without AMF inoculation. Bar hatching highlights treatments in which the naturally occurring AMF were missing after γ-irradiating the soil. Bars show mean values and associated standard errors of five experimental replicates. The significances (***, p<0.001) of the effect of green manure addition and mineral Zn fertilization from a fixed factor four-way analysis of variance are indicated above the bars. For full statistical details see Table 2.

The Zn from the added green manure recovered in the aboveground biomass (Zn_rec_gm_) ranged between 2.5% and 4.1% for red clover, and between 1.4% and 2.7% for sunflower (Figure 4). These Zn recoveries from the two types of green manures differed significantly across all treatments (F_1,79_ = 72.25, p<0.001). Mineral Zn fertilization lowered Zn_rec_gm_ for both forms of green manure (F_1,64_ = 22.31, p<0.001, Figure 4 & Table 2), as did soil γ-irradiation (F_1,64_ = 8.85, p<0.01). The recovered Zn fraction from sunflower residues was also reduced by the inoculation of *C. claroideum* when also mineral Zn fertilizer was applied (F_1,18_ = 10.84, p<0.01; Figure 4).

The amount of Zn in the aboveground biomass derived from green manure (Zn_dgm_) ranged between 4.5 µg Zn (kg soil)^−1^ in the treatment of combined soil γ-irradiation, mineral Zn fertilization, and addition of green manure of red clover, and 11 µg Zn (kg soil)^−1^ in the treatment with green manure of sunflower but without mineral Zn fertilization, soil γ-irradiation, and AMF inoculation (Table 3). The amount of plant Zn derived from green manure of sunflower (Zn_dgm_) was consistently higher than that derived from red clover (F_1,64_ = 39.92, p<0.001; Tables 2 & 3).

**Figure 5:**
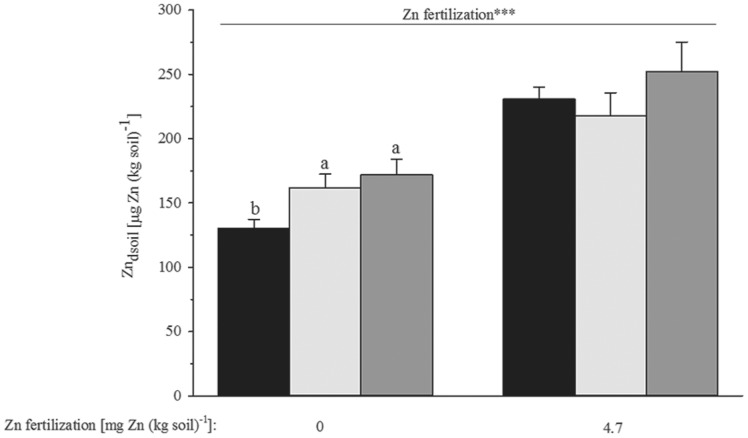
Zinc (Zn) uptake from soil to the aboveground biomass of bread wheat grown in a calcareous soil after addition of two types of green manure. The Zn taken up from soil either originated from the native soil Zn pool or from soil as well as applied ZnSO_4_ fertilizer. Bar filling denoting the different green manure addition treatments is the same as in Figure 1. Bars show mean values and associated standard errors of five experimental replicates. The significance (***, p<0.001) of the effect of mineral Zn fertilization from a fixed factorial four-way analysis of variance is indicated above the bars. Different letters indicate statistical differences of separate least significant difference tests at p<0.05 for the different green manure addition treatments within the different Zn fertilization levels. For full statistical details see Table 2.

The amount of Zn derived from the native soil Zn pool (Zn_dsoil_) was significantly increased with green manure addition, when no ZnSO_4_ was applied (F_2,96_ = 3.50, p = 0.034, Table 2 & Figure 5). This apparent soil Zn mobilization was more pronounced for green manure produced from sunflower than from red clover (Figure 5). Under conditions of co-application of ZnSO_4_, the effect of green manure addition was, however, variable and weak and statistically not significant. There was also no significant effect of γ-irradiation, nor any effect by the non-indigenous *C. claroideum* inoculant on the total amount of Zn derived from soil and mineral Zn fertilizer, either (Table 2).

### Changes in DTPA-extractable Zn from soil, total N and pH in soil

The concentration of DTPA-extractable Zn from soil measured after plant harvest was significantly higher in the treatments with green manure than without green manure (F_1,96_ = 169, p<0.001). Application of mineral Zn fertilizer also significantly increased DTPA-extractable Zn (F_2,96_ = 85.2, p<0.001; Tables 2 & 3). There was a highly significant correlation between the total Zn taken up into the aboveground biomass (Y) and the DTPA-extractable Zn concentration in the soil (X) according to a double reciprocal regression model [Y = 1/(0.004 + 2.05/X)] with r^2^ = 0.56 and p<0.0001.

Green manure addition slightly decreased soil pH, independently of the soil γ-irradiation treatment (F_2,96_ = 139.4, p<0.001; Table 4). The total soil N content was higher at harvest in the treatments that received green manure than in those that did not (F_2,96_ = 321.7, p<0.001, Table 2 & 4).

**Table 4:** Total soil nitrogen (N) concentration and pH in water at wheat harvest.

Soil	Green manure	Total N (g kg^−1^)	pH (H_2_O)
		(Mean ± SE)	(Mean ± SE)
**Non-irradiated**	**None**	0.55±0.02^B^	7.75±0.03^A^
	**+ Red clover**	0.87±0.02^A^	7.50±0.02^B^
	**+ Sunflower**	0.88±0.02^A^	7.53±0.01^B^
**γ-irradiated**	**None**	0.63±0.01^b^	7.64±0.02^a^
	**+ Red clover**	0.89±0.02^a^	7.46±0.01^b^
	**+ Sunflower**	0.96±0.02^a^	7.48±0.01^b^

DTPA: diethylene-triamine-penta-acetic acid

The values represent the means and associated standard errors (SE) of 20 experimental units. Different superscript letters of the same type following the SE indicate statistical difference among the means of the three green manure treatments within each soil γ-irradiation treatment at p<0.05, according to least significance difference tests.

## Discussion

The results confirmed our first prediction, because green manure addition to soil increased Zn uptake by bread wheat. The findings, however, did not unequivocally support our second prediction, since utilization of the applied mineral Zn fertilizer by the plants did not improve in response to green manure addition. Also our last prediction that inoculation of a non-indigenous AMF strain would improve Zn transfer from soil to plants was not confirmed, not even in AMF- and thus competitor-free soil after removal of naturally occurring AMF by soil γ-irradiation. These two later findings appear to be related to the fact that Zn availability in the studied soil was neither limiting plant growth, nor yield.

In agreement with previous studies [43], [44], we observed a clear increase in grain Zn concentration after application of mineral Zn fertilizer. However, application of mineral Zn fertilizer did neither translate into increased plant growth, nor higher grain yields, showing that Zn availability was not the primary plant growth-limiting factor in this soil. Sadeghzadeh [42] reports DTPA-extractable Zn levels below 0.6 mg Zn (kg soil)^−1^ to limit wheat growth in calcareous soil. Since our soil contained 0.47 mg Zn (kg soil)^−1^ and application of mineral Zn fertilizer did not promote wheat growth, it appears that the threshold for growth-limitation of soil Zn concentration may even be lower, although Zn uptake can obviously be raised by application of mineral Zn fertilizer.

### Mixing green manure to soil increased Zn uptake by wheat via Zn mobilizing effects

The observed increase in Zn uptake from the native soil Zn pool after green manure addition could originate from changes to the soil conditions, root growth, and/or plant physiology. Only addition of green manure to the soil increased the DTPA-extractable Zn from 0.47 to 0.5 or 2 mg Zn (kg soil)^−1^, when green manure of red clover and sunflower were added, respectively. Combining ZnSO_4_ application with green manure addition to soil increased the DTPA-extractable Zn concentration even further, namely up to 3.8 mg Zn (kg soil)^−1^ when red clover was used and up to 5.0 mg Zn (kg soil)^−1^ when sunflower was used, respectively (Table 3). We thus can conclude that the lack of a plant growth response to application of mineral Zn fertilizer (Figure 1A&B) must be explained by an alternative plant growth-limiting factor and not the >0.6 mg Zn (kg soil)^−1^ of DTPA-extractable Zn [42].

The larger increase in DTPA-extractable soil Zn in response to addition of green manure of sunflower than such of red clover (Table 3) could have been due to the Zn-richer green manure of sunflower than the green manure of red clover. For production of the green manure of sunflower, but not that of red clover, only leaf laminae were used, which could explain the higher mineral nutrient concentrations of it (Table 1). Moreover, the relatively young age (54 day) and bigger sunflower than red clover seeds with own nutrient reserves may have further contributed to nutrient concentration differences of the two types of green manure (Table 1), translating into differences in plant Zn uptake.

There may have been further co-nutritional or root- and microbial growth-mediated Zn uptake stimulation, because the green manure produced of sunflower was also richer in N than that of red clover (Table 1). The green manure of the leaf laminae of the well N-supplied sunflower plants could have released N during its decomposition and stimulated root growth and possibly the synthesis of Zn facilitating N-rich plant and microbial compounds. Elevated root and microbial exudation and necromass in response to green manure of sunflower may also explain the higher DTPA-extractable Zn concentrations of the soil and increased plant Zn uptake. Root- and microbe-derived organic acids, amides and (phyto-) siderophores may have chelated and thereby mobilized Zn for uptake by the wheat plants. More N released from the decomposing green manure of more N-rich sunflower than red clover material (Table1) may have supported the N-demanding biosynthesis of Zn chelating compounds [8], [14], [45].

Elevated root and microbial activity in response to green manure addition may further have lowered soil pH (Table 4) and thereby mobilized soil Zn. Similarly, stimulation of nitrification upon higher N inputs with the green manure of sunflower than that of red clover and thus release of protons into the soil solution may have raised Zn bioavailability in soil for uptake by the mycorrhized wheat plants.

All in all, the effectiveness of green manure addition to soil to release Zn from the native soil Zn pool for plant uptake was remarkable (Figure 5). This stimulatory effect on plant Zn uptake diminished, however, when additionally ZnSO_4_ fertilizer was applied. This is probably, because the applied ZnSO_4_ fertilizer introduced already a high amount of Zn, compared to the amount solubilized by chelating compounds released upon addition of green manure, reducing Zn mobilization form soil.

### Inoculation of a non-indigenous AMF strain did not increase Zn uptake in wheat

A previous experiment (Forough Aghili, unpublished) with the same soil and wheat cultivar and additional analyses on samples of this experiment (Anouk Guyer, unpublished data) showed that the roots of the wheat plants were colonized by naturally occurring AMF of this soil. 1.6 kb-long ribosomal DNA sequences confirmed root colonization by the inoculated Swiss strain of *C. claroideum* of the wheat plants raised in γ-irradiated soil. We suspect that maladaptation of the AMF inoculant to the living conditions in the experimental soil [46] must have compromised symbiotic Zn acquisition by wheat. The inoculated AMF was originally isolated from Swiss arable soil, not calcareous, and with a loamy texture, a lower, near neutral pH, and much richer in organic matter and bioavailable nutrients [47] than the study soil here. The improved Zn recovery from green manure of sunflower after application of mineral Zn fertilizer by the plants inoculated with the foreign AMF strain points at possibly complex soil-root-microbe interactions beneficial to mycorrhized plants. Lack, or just minor positive effects by the non-indigenous AMF inoculant should draw our attention to the fact that AMF inoculants can not be *a priori* considered effective in assisting crop plants to take up Zn from soil when introduced to soils of different physicchemical and biological properties.

γ-Irradiation has been shown to modify the physicochemical soil properties to some degree [48], [49]. However, we believe that this did not invalidate our results. While the increased biomass (Figure 1) and N uptake (Figure 2) of the wheat plants grown in γ-irradiated soil can probably be related to released N from decomposing microbial necromass [49], the magnitude of this effect was certainly much smaller than that by the N added with the green manures (Figure 2, Table 2). Likewise, the levels of dissolved organic compounds [48] must have risen in response to γ-irradiation, but again this effect must have been considerably smaller than that caused by mixing 0. 4% (w/w) finely ground green manure to soil. Therefore, following Thompson [33], and until a mycorrhiza symbiosis-defective wheat mutant becomes available, we see no better approach than using γ-irradiation for the study of the influence of entire natural AMF assemblages on plant Zn uptake from real arable soil.

The finding of higher grain and aboveground Zn concentrations in plants raised on native than γ-irradiated soil (Figure 3) points at no Zn uptake stimulatory effect by elevated levels of dissolved organic compounds [48] and N after soil γ-irradiation [49]. The generally increased Zn tissue concentration after addition of green manure to soil (Figure 3) and higher Zn recovery from less N- and Zn-rich green manure of red clover than more N- and Zn-rich green manure of sunflower point at considerable influences by C- rather than N-limited saprotrophic soil microbes on Zn uptake of mycorrhized bread wheat plants. Being obligate biotrophs, AMF rely on saprotrophs for remobilization of mineral nutrients from decomposing green manure that they may as soon as available take up and then possibly deliver to their host plants as observed for the Zn from the green manure of sunflower (Figure 4). Much higher Zn recovery from the Zn- and N-poorer green manure of red clover than the Zn-richer and N-poorer green manure of sunflower (Figure 4) points at faster decomposition of the earlier than latter and thus also different speeds in nutrient recycling from different green manures. However, the two types of green manure appear not to have differed in Zn mobilization from soil (Figure 5).

Native as opposed to γ-irradiated soil after back-addition of an AMF propagule-free microbial filtrate of a water suspension of living soil does certainly not only differ in AMF occurrence. Besides the physicochemical changes discussed above [49], largely stochastic epidemic population growth of saprophytic organisms must occur [35]. Therefore, importance of fungi and microbes conferring similar nutritional benefits to plants as AMF [50] may increase in absence of otherwise naturally occurring AMF and much reduced population sizes of most of other just re-introduced soil microbes. Microbial nutrient competition with plants [34] may be particularly harsh when microbial population growth after inoculation coincides with release of nutrients from new microbial necromass and freshly applied mineral fertilizer, as it must have occurred in the γ-irradiated control soil of this experiment. The re-introduced soil microbes in the filtrate of the soil suspension and the newly inoculated foreign AMF inoculant may thus have mainly sequestered Zn and other nutrients in their newly formed biomass in γ-irradiated soil [34]. This would further explain why the inoculated foreign AMF strain did not assist much in plant Zn uptake.

In summary, this study advanced our knowledge on possible agronomic practices to biofortify cereal grains with Zn. i) It showed that addition of readily decomposable N-rich green manure to calcareous soil can raise grain Zn concentration of bread wheat to levels approaching those of only ZnSO_4_ fertilizer-fed wheat plants; ii) The study showed that combined addition of N-rich green manure and ZnSO_4_ fertilizer can considerably raise grain Zn concentration; iii) Mixing green manure to the soil showed that the native soil Zn pool can contribute considerably to total Zn accumulation in the aboveground plant biomass even when no additional mineral Zn fertilizer had been applied to the soil; iv) Lastly, the study emphasizes that care should be taken when trying to utilize AMF inoculants for supporting crop plants in their Zn uptake, when the fungi are not adapted to the prevailing soil conditions.
